# Tactile cortical responses and association with tactile reactivity in young children on the autism spectrum

**DOI:** 10.1186/s13229-021-00435-9

**Published:** 2021-04-01

**Authors:** Svenja Espenhahn, Kate J. Godfrey, Sakshi Kaur, Maia Ross, Niloy Nath, Olesya Dmitrieva, Carly McMorris, Filomeno Cortese, Charlene Wright, Kara Murias, Deborah Dewey, Andrea B. Protzner, Adam McCrimmon, Signe Bray, Ashley D. Harris

**Affiliations:** 1 Department of Radiology, Cumming School of Medicine, University of Calgary, 2500 University Drive NW, Calgary, AB T2N 4N1 Canada; 2 Department of Clinical Neuroscience, Cumming School of Medicine, University of Calgary, Calgary, AB Canada; 3 Child and Adolescent Imaging Research (CAIR) Program, University of Calgary, Calgary, AB Canada; 4 Department of Paediatrics, Cumming School of Medicine, University of Calgary, Calgary, AB Canada; 5 Alberta Children’s Hospital Research Institute, University of Calgary, Calgary, AB Canada; 6 Hotchkiss Brain Institute, University of Calgary, Calgary, AB Canada; 7 The Mathison Centre for Mental Health Research and Education, University of Calgary, Calgary, AB Canada; 8 Community Health Sciences, Cumming School of Medicine, University of Calgary, Calgary, AB Canada; 9 Werklund School of Education, University of Calgary, Calgary, AB Canada; 10 Department of Psychology, Faculty of Arts, University of Calgary, Calgary, AB Canada

**Keywords:** Somatosensory-evoked potentials, ERP, EEG, Adaptation, Tactile stimulation, Tactile sensitivities, Autism, Children

## Abstract

**Background:**

Unusual behavioral reactions to sensory stimuli are frequently reported in individuals on the autism spectrum (AS). Despite the early emergence of sensory features (< age 3) and their potential impact on development and quality of life, little is known about the neural mechanisms underlying sensory reactivity in early childhood autism.

**Methods:**

Here, we used electroencephalography (EEG) to investigate tactile cortical processing in young children aged 3–6 years with autism and in neurotypical (NT) children. Scalp EEG was recorded from 33 children with autism, including those with low cognitive and/or verbal abilities, and 45 age- and sex-matched NT children during passive tactile fingertip stimulation. We compared properties of early and later somatosensory-evoked potentials (SEPs) and their adaptation with repetitive stimulation between autistic and NT children and assessed whether these neural measures are linked to “real-world” parent-reported tactile reactivity.

**Results:**

As expected, we found elevated tactile reactivity in children on the autism spectrum. Our findings indicated no differences in amplitude or latency of early and mid-latency somatosensory-evoked potentials (P50, N80, P100), nor adaptation between autistic and NT children. However, latency of later processing of tactile information (N140) was shorter in young children with autism compared to NT children, suggesting faster processing speed in young autistic children. Further, correlational analyses and exploratory analyses using tactile reactivity as a grouping variable found that enhanced early neural responses were associated with greater tactile reactivity in autism.

**Limitations:**

The relatively small sample size and the inclusion of a broad range of autistic children (e.g., with low cognitive and/or verbal abilities) may have limited our power to detect subtle group differences and associations. Hence, replications are needed to verify these results.

**Conclusions:**

Our findings suggest that electrophysiological somatosensory cortex processing measures may be indices of “real-world” tactile reactivity in early childhood autism. Together, these findings advance our understanding of the neurophysiological mechanisms underlying tactile reactivity in early childhood autism and, in the clinical context, may have therapeutic implications.

**Supplementary Information:**

The online version contains supplementary material available at 10.1186/s13229-021-00435-9.

## Introduction

Autism spectrum (AS) describes a heterogeneous neurodevelopmental condition diagnosed based on social communication deficits and restricted, repetitive behaviors [6]. However, the presence of sensory difficulties has recently been recognized as a core feature of autism (in the DSM-5 [6]), consistent with estimates suggesting that over 90% of individuals on the autism spectrum^1^ show unusual behavioral reactions to sensory stimuli that persist across age [11, 53, 85]. However, sensory difficulties in autism are highly heterogeneous, including both hyper- and hypo-reactivity that limit everyday functioning [11, 53, 84, 85]. Understanding the neurophysiological processes underlying sensory difficulties may yield crucial insights into the condition, and may also have clinical implications for improving therapies and creating sensory-friendly environments for individuals with autism.

Interest in sensory processing differences in autism has surged, with many studies focusing on auditory and visual modalities, likely due to their relevance for communication (for review see [59]). Far less work has been done to understand unusual reactions to tactile stimuli in autism (e.g., avoiding light touch as occurs with grooming and from clothing or seeking out pressure stimuli), despite the central role of touch in early development of social, communication and motor abilities [17, 84]. It is during the early years of life that tactile difficulties emerge (< age 3 years) [53, 61] and may exacerbate the core social communicative and behavioral features observed in autism [38, 74].

To date, neuroimaging studies in autism have identified differences in somatotopic mapping [24] and reduced evoked responses to tactile stimulation in somatosensory cortex [19, 40, 60]. However, other studies have reported normal [18, 23, 30, 42, 51] or even enhanced [47, 50, 64] somatosensory responses. Neural responses to touch have also been related to parent- or self-reported tactile features in older children and adolescents with autism [18, 60].

Another area that impacts tactile processing is how neural responses adapt to repeated stimulation. This neurophysiological process, whereby the neural response strength decreases with repeated stimulation, is thought to “filter” out invariant stimuli after constant exposure and conserve attentional resources [90, 91]. In the tactile domain, behavioral [72, 83, 86] and imaging [43] observations suggest reduced adaptation in autism, including in infants at elevated likelihood of autism [67], but see [16, 44]. Taken together, these findings suggest that altered inhibitory function, in line with the mechanistic proposal of imbalance between excitation and inhibition [77] could explain some of the features of tactile reactivity in autism.

However, work to date has focused mainly on adults or children older than 6 years due to practical and methodological challenges associated with testing young children. This leaves a gap in our knowledge of the neural basis of tactile processing in early childhood autism. Understanding tactile cortical responses in this time period is of particular importance due to the early emergence of sensory difficulties in autism [53, 61] and the emphasis on early intervention in promoting better quality-of-life outcomes for autistic children [20, 52, 76, 78].

Thus, the aim of this study was to assess tactile cortical processing in young children with autism aged 3–6 years using electroencephalography (EEG) and to determine if properties of somatosensory-evoked potentials (SEPs) are associated with “real-world” parent-reported tactile reactivity. To avoid propagating inconsistent or imprecise terminology in the field of sensory processing [79], we will use “reactivity” to refer to an individual’s reaction to sensory input that involve emotional and behavioral disruptions, as assessed by parent-reports [81]. EEG is an ideal tool for challenging pediatric populations (e.g., easy to apply, no loud noises, less sensitive to movement than MRI) and provides insight into cortical processes with excellent temporal resolution, thus allowing us to understand which stages of tactile processing (early or later stages) might be aberrant in autism. In particular, early (P50, N80) and mid-latency (P100, N140) SEP responses are thought to reflect unconscious and conscious processing of stimulus properties, while later responses reflect the perceptual and cognitive processing of stimuli (e.g., P300) (e.g., [2, 31, 54, 80]). Based on mixed previous findings [18, 19, 30, 40, 42, 60], we expected SEP measures to be different in young children with autism compared to neurotypical (NT) children, but did not hypothesize a direction for differences. Further, given links between tactile cortical responses and tactile behavioral features in autism [18, 60], we hypothesized that SEP measures would be associated with parent-reported tactile reactivity.

## Methods

### Participants

Thirty-three young children with autism aged 3–6 years were initially recruited from the Owerko Neurodevelopmental Disorder Recruitment database and the local community. All autistic children had a prior clinician diagnosis, which often included the administration of the Autism Diagnostic Observation Schedule (ADOS) [57]. Clinician diagnosis was supported by parent reports on the Social Responsiveness Scale, Second Edition (SRS-2), a quantitative measure of clinical autistic traits [22]. When an autistic child scored below the cut-off on the SRS-2 (≤ 59 T), an ADOS was administered by a research-reliable rater to confirm diagnosis. Exclusion criteria included known genetic etiology of autism (e.g., Fragile X syndrome, tuberous sclerosis), seizures at the time of study entry, a history of major head trauma or loss of consciousness of > 5 min and/or neurologic disease. Four children with autism had also been diagnosed with attention-deficit/hyperactivity disorder (ADHD), one global developmental delay (GDD) and one was born prematurely at 27 weeks gestational age. Two children were receiving medication used to treat ADHD (one received Strattera and Intuniv, and one received Vyvanse). These medications were withheld for at least 24 h prior to the study visit (when possible, and with parental consent). Excluding all autistic participants with comorbidities and pre-term birth (*N* = 6) did not change the results reported here and so these participants were included in the analyses.

Forty-five age- and sex-matched NT children were recruited using the Healthy Infants and Children Clinical Research Program (HICCUP) and community advertisements. NT participants were excluded if they had a history of neurological, psychiatric or neurodevelopmental disease, a history of major head trauma or loss of consciousness of > 5 min, were born prematurely (< 37 weeks), were using psychotropic medications, or scored above the cut-off on the SRS-2. There were no significant differences between groups in age, sex or handedness (as assessed by a parent questionnaire adapted from [48]) (see Table 1).

**Table 1:** Characteristics of study participants

	NT	AS	Statistics
*N*	41	28	
Age [years]	5.3 ± 1.1	5.4 ± 1.1	*t*_(67)_ = − 0.60, *p* = 0.553
Sex (M:F)	28:13	22:6	*Χ*^*2*^ = 0.88, *p* = 0.348
Handedness (R:L:A)	38:2:1	27:0:1	*Χ*^*2*^ = 1.46, *p* = 0.481
SRS-2T-score	45.1 ± 5.8	79.3 ± 12.5	***t***_**(34.9)**_** = **− **13.60, p < 0.001**
Non-verbal IQ	106.4 ± 14.8	95.8 ± 21.9	***t***_**(33.0)**_** = 2.92, p = 0.006**
Overall tactile reactivity	13.6 ± 3.9	25.4 ± 9.1	***F***_**(1,65)**_** = 51.43, p < 0.001**
Tactile hyper-reactivity	5.7 ± 2.2	10.6 ± 4.6	***F***_**(1,65)**_** = 39.3, p < 0.001**
Tactile hypo-reactivity	7.9 ± 2.7	14.55 ± 7.1	***F***_**(1,65)**_** = 27.7, p < 0.001**

All participants had normal or corrected-to-normal vision. Group differences in sex and handedness between children on the autism spectrum (AS) and a neurotypical (NT) comparison group were assessed using chi-square test. Significant effects are indicated in bold

General cognitive ability of all children was measured using the brief version of the Wechsler Non-Verbal (WNV) Scale of Ability [65], which allows for the assessment of individuals with limited language skills. In all but 3 autistic children, who did not understand and/or respond to non-verbal instructions, a non-verbal IQ estimate was obtained (Table 1). While we were unable to obtain IQ estimates, we were still able to acquire EEG data from these children because this did not require semantic or pragmatic comprehension nor an overt response to the tactile stimulation.

The study was approved by the University of Calgary Conjoint Health Research Ethics Board (REB16-0576). Written informed consent in accordance with the Declaration of Helsinki was obtained from a parent/guardian of each child who themselves assented to testing.

### Tactile reactivity measures

Parents completed the Child Sensory Profile 2 (CSP-2; [35]), a standardized parent-report questionnaire that measures sensory processing patterns in everyday life. For each item, parents were asked to rate their child’s reaction to a sensory experience on a 5-point Likert scale ranging from ‘Almost Never’ (1) to ‘Almost Always’ (5). While the CSP-2 addresses multiple sensory domains (e.g., visual, auditory, oral), this study focused exclusively on the tactile domain. We derived three measures representing overall tactile reactivity as well as tactile hyper- and hypo-reactivity. Specifically, scores for all questions related to the tactile domain were summed to yield a tactile reactivity measure (CSP-2 questions 16–26). To explore whether tactile hyper- or hypo-reactivity relate to SEP measures, scores for questions related to sensitivity and avoiding (e.g., “My child shows an emotional or aggressive response to being touched.”) were summed to yield a tactile hyper-reactivity measure (CSP-2 questions 16–20), while scores for questions related to registration and seeking (e.g., “My child touches people and objects more than same-aged children.”) were summed to yield a tactile hypo-reactivity measure (CSP-2 questions 21–26). Please note that each of these two behavioral profiles were made up of different CSP-2 quadrants within the tactile domain and do not reflect opposite ends of the same scale. Thus, for all three tactile reactivity measures, higher scores indicate more sensory difficulties.

### Procedure

**Fig. 1:**
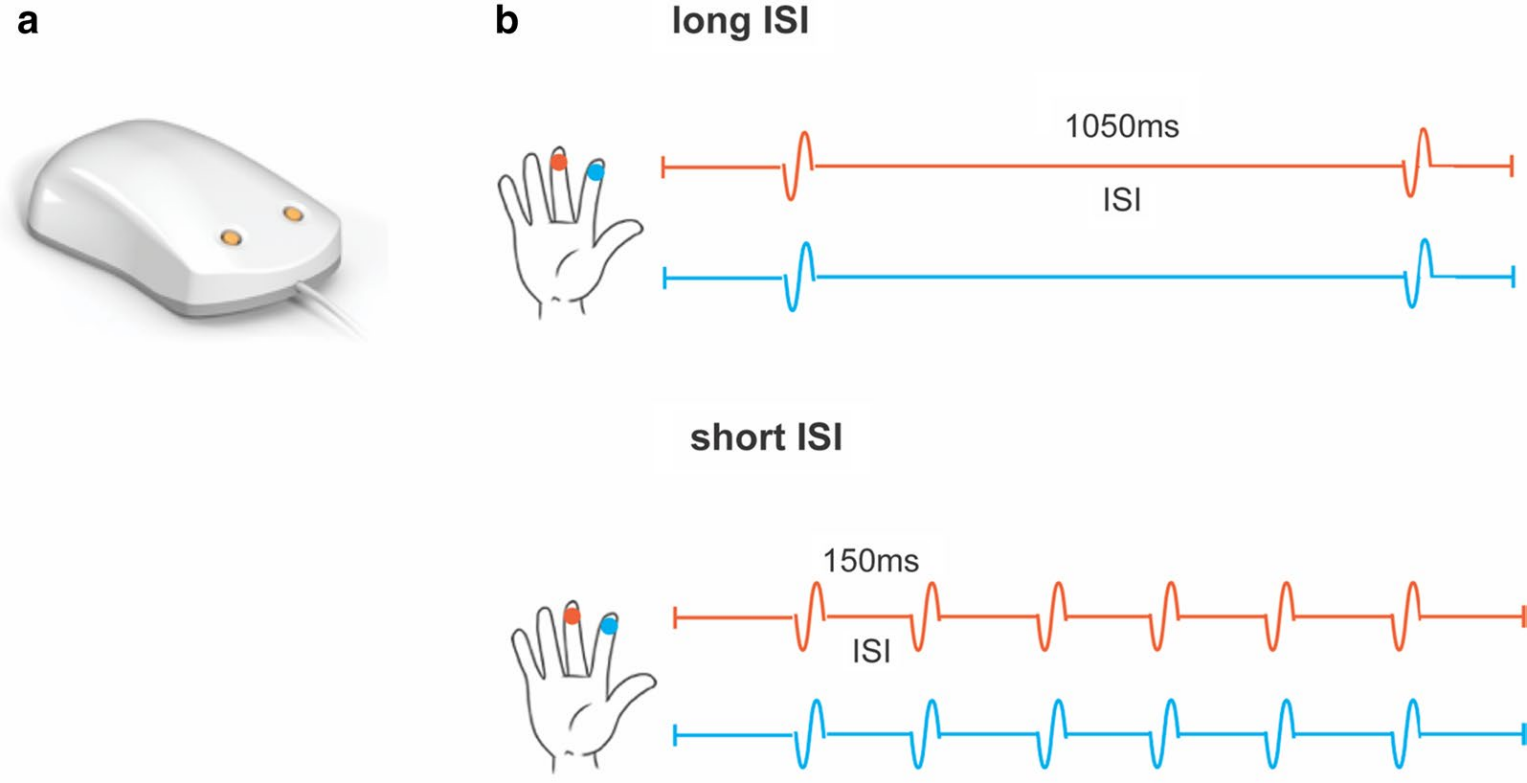
Tactile stimulation paradigm. **a** A Brain Gauge two-digit vibrotactile stimulator was used for stimulus generation. **b** Schematic of the passive tactile stimulation. Trains of 6 tactile stimuli were delivered simultaneously to the right-hand digit 2 and digit 3. In the long ISI condition stimuli were delivered further apart in time (ISI of 1050 ms), while in the short ISI condition stimuli were presented closer together (ISI of 150 ms) which typically leads to a reduction in somatosensory cortex response. Each stimulus train was separated from the next by 5 ± 0.5 s

Electroencephalography (EEG) was recorded while participants received passive tactile stimulation to the fingertips (index and middle finger) of their right hand (Fig. 1). Prior to the testing session, parents were interviewed regarding their child’s communication, behavior and interests in order to individually tailor the testing environment and session to be as comfortable as possible for each child. A video of a child participating in EEG testing was sent to parents to review with their child prior to the session (YouTube: “What is a research EEG like?”). During the testing session, behavioral strategies were used to support EEG data acquisition, supported by an occupational therapist (C.W.). These strategies included parents as partners (e.g., working with parents to achieve child participation through shared decision-making), visual supports (e.g., pictures used to communicate and interact with autistic children), desensitization (e.g., gradual exposure to a feared or aversive stimulus until emotional response is tolerable), and individualized reinforcement (e.g., rewards that are motivating for the specific individual). To further increase compliance during the EEG recording [39, 88], participants watched a movie of their or their parent’s choice on a 15-inch HD monitor (Dell Inspiron 15 3000 Series, display dimensions 1366 × 768 mm, resolution 1366 × 768 pixels, refresh rate 60 Hz) with the volume adjusted to each participant’s personal preference level. Importantly, we have previously shown that movie-watching does not modulate properties of somatosensory-evoked potentials (SEPs) [36]. Breaks were included as required, including briefly pausing the delivery of stimuli to suit child comfort. Together, these strategies enabled successful EEG data collection from more than 80% of children on the autism spectrum and 95% of NT children in this challenging age range, including those with low cognitive and/or verbal abilities who are often excluded from neuroimaging research (see Results section for more details).

#### Tactile EEG task

Participants received passive tactile stimulation to their right index and middle fingers simultaneously. No behavioral responses to the stimuli were required. Somatosensory mechanical stimuli were generated using a customized Brain Gauge two-digit vibrotactile stimulator (Cortical Metrics, North Carolina, USA) (Fig. 1a). Stimulus delivery was controlled by a computer running Presentation software (Neurobehavioral Systems, Berkeley, CA, USA). All stimuli were suprathreshold (frequency 25 Hz, amplitude 300 μm, duration 40 ms) and delivered to the glabrous skin of the fingertips using cylindrical probes (5 mm in diameter). Tactile stimulation consisted of four successive blocks with fifty repetitions of a 6-stimulus train in each block (300 stimuli in total in each block). We chose a block-presentation paradigm, as it has been suggested to be more effective in eliciting adaptation than paired paradigms [7]. Each train of 6 stimuli was separated from the next by an interval of 5 ± 0.5 s (measured from the last stimulus in a train to the first stimulus of the preceding train). Within each block, the inter-stimulus interval (ISI) within the trains of tactile stimulation was constant and set to either 1050 ms or 150 ms (Fig. 1b). The order of the long (1050 ms) and short (150 ms) ISI blocks was counterbalanced. Shorter ISIs, in which stimuli are presented close together in time, typically lead to a reduction in the cortical response amplitude, hereafter referred to as adaptation [8, 9, 87]. Thus, while the 1050 ms ISI was used to assess somatosensory processing of individual stimuli, the 150 ms ISI allowed us to assess somatosensory adaptation. The duration of the somatosensory stimulation was ~ 15 min and an experimenter monitored each child throughout to ensure compliance and provided verbal support when necessary.

#### EEG data acquisition

Scalp EEG was recorded at 1000 Hz using a 64-electrode (equidistant) geodesic sensor net (Electrical Geodesic Inc., Oregon, USA) soaked in an electrolyte solution. All electrodes were spaced evenly and symmetrically to cover the scalp. The impedance level was kept below 50 kΩ and the EEG signal was referenced to Cz during recording. The timing of the first tactile stimulus in a train was marked in the simultaneous EEG recording, with separate markers for each ISI condition (long, short).

### EEG data analysis

#### Pre-processing

EEG data pre-processing and analysis were performed using EEGLAB (version 14) [29], ERPLAB Toolbox (version 7) [56] and additional scripts written in Matlab (version R2017b). The raw EEG signal was filtered using a high-pass filter at 0.1 Hz and a low-pass filter at 45 Hz, downsampled to 250 Hz, and re-referenced to the average signal of all electrodes. Excessively noisy EEG electrodes (mean 1 ± 2 electrodes, range 0–6 electrodes) were removed and interpolated prior to re-referencing so as to not include excessive noise in the common average. Ocular artifacts were corrected by applying independent component analysis (ICA) as implemented in EEGLAB (excluding the interpolated electrodes). Artifact independent components (ICs) were visually identified using SASICA [21] as a guideline. An average of 3.2 ± 1 ICs (range 2–6 ICs) ocular artifacts were removed, and the number of ICs did not differ between groups [*F*_(67))_ = − 1.62, *p* = 0.111].

The continuous EEG signal was epoched from − 50 to 500 ms relative to stimulus onset (0 ms) and baseline corrected using the 50 ms pre-stimulus period. While a 50 ms baseline is relatively short, we verified that our results remain unchanged when using a longer 100 ms pre-stimulus priod (Additional file 1: Table S3). EEG trials were visually inspected and trials containing residual artifacts (e.g., due to movement or talking) were removed. The average number of trials used to compute SEPs was 210 ± 34 for the AS group and 228 ± 24 for the NT group. As expected, the AS group had a lower trial retention rate for both the long ISI [*t*_(39.39)_ = 2.54, *p* = 0.015] and short ISI [*t*_(67)_ = 2.19, *p* = 0.032] trials compared to the NT group. In all cases, a minimum of 150 trials were evaluable (AS: range 151–258; NT: range 187–277). Trial retention was unrelated to IQ or age (both *p* > 0.5). From our observations and previous findings [34], it appears that the participant’s affective state influenced EEG trial retention (e.g., children who were upset or irritable prior to the start of testing were less compliant). To verify that trial retention rate did not influence our findings, we re-ran our analyses including the number of remaining trials as a covariate, which did not change the results reported here (see Additional file 1: Table S1 and S2).

#### SEP analysis

The artifact-free EEG data were averaged over trials and participants. Visual inspection of the grand-average topography and SEP traces for both groups showed early and mid-latency SEP responses P50 (30–55 ms), N80 (55–80 ms), and P100 (80–125 ms), as well as later responses N140 (150–210 ms) and P300 (270–300 ms; also called late positive component), which were all most prominent at electrodes situated above the somatosensory cortex contralateral to the stimulated fingers [2–4, 15, 33, 45, 69]. Given that the somatosensory cortex has been suggested to be functionally different in individuals with autism [24], individual regions of interest (ROIs) were selected based on electrodes that showed a major positive peak in the 30–55 ms and 80–125 ms time windows. In addition, regions beyond the somatosensory cortex, such as the bilateral frontal lobes, have been shown to be active during the later stages of tactile processing [2, 4, 5, 32]. For this reason, two additional responses, P190 (150–240 ms) and N300 (280–400 ms), were evaluated from a ROI over the bilateral frontal cortex. For each participant, 5 electrodes were selected and averaged for each ROI (somatosensory and frontocentral) from SEP data averaged over the respective time windows. The same ROIs were used for all analyses for that participant. The topographic plot in Fig. 3a , b (top panel) shows a strong overlap across participants in the electrodes selected. Selected time windows applied to all participants and ISIs, and were not adjusted individually.

For each individual participant, the peak latency and mean amplitude for each SEP response was derived from the respective time windows and individual ROIs to investigate potential differences in the speed and strength of processing between groups.

In addition, we derived the mean amplitude difference between the long (ISI of 1050 ms) and short (ISI of 150 ms) ISI to characterise adaptation of SEP responses with repetitive stimulation. Note that a quantitative assessment of adaptation was only possible for the early and mid-latency SEP responses (P50, N80, P100) as the time window for later SEP responses overlapped with the SEP to the subsequent stimulus in the short ISI.

### Statistical analysis

Statistical analyses were performed using SPSS (IBM SPSS Statistics, Armonk, NY, USA) and custom-written Matlab routines. Effects of group (AS, NT) on tactile reactivity measures (overall tactile reactivity, hyper- and hypo-reactivity) and properties of SEPs (peak latency and mean amplitude) were assessed using analysis of covariance (ANCOVA), with ‘group’ (2 levels: NT, AS) as a between-participants factor. To assess group differences in the reduction in SEP amplitude with repetitive stimulation (adaptation), repeated-measures ANCOVAs with ‘ISI’ (2 levels: long ISI, short ISI) as within-participants factor and ‘group’ as between-participants factor were used, in line with previous studies utilising block-presentation paradigms [8, 87]. A significant interaction between ‘ISI’ and ‘group’ would suggest differences in the amount of adaptation between NT and AS groups. All analyses were controlled for age and sex given their influence on somatosensory cortical processing [1, 14, 68, 93]. Whenever group differences were found, additional analyses controlling for non-verbal IQ were conducted. This was because lower IQ was clearly part of the autism phenotype in our data, so that including IQ as a covariate might have reduced the power to detect group differences.

All variables were tested for normality (using the Kolmogorov–Smirnov test). In the presence of non-normality data were bootstrapped with replacement (1000 bootstrapped samples for AS and NT groups of their respective sample size) to estimate the *p* value of the test statistic. Mauchly’s test of sphericity was used to assess homogeneity of variance and a Greenhouse-Geiger correction was applied whenever Mauchly’s test indicated a lack of sphericity.

To examine associations between neural measures (latency, amplitude, and adaptation of SEP responses) and parent-reported tactile reactivity, we used general linear models of the whole sample that included group (AS, NT) by tactile reactivity interactions, along with main effects, and age and sex as covariates. Significant statistical interactions, denoting group differences in associations, were then followed up with partial correlations to test associations within groups separately. Multiple comparisons were controlled for using the false discovery rate (FDR), as described by Benjamini and Hochberg [13]. A *p*_corr_ < 0.05 was used after correcting for multiple comparisons across SEP responses within each ROI (contralateral somatosensory ROI: 5; frontocentral ROI: 2). Uncorrected *p* values are reported as well. All data presented in the text and figures are represented as mean ± SD unless stated otherwise.

## Results

### Participant characteristics

Participant characteristics are shown in Table 1. The final sample included 28 autistic children and 41 NT children aged 3–6 years, which were matched for age, sex, and handedness (statistics and *p* values are summarized in Table 1). The male-to-female ratio of participants with autism was 3.7:1. Of the 33 autistic children recruited, 5 children did not tolerate the EEG procedure. Of the 45 NT children recruited, 2 children did not tolerate the EEG procedure and another 2 children were excluded as they scored above the cut-off for autism on the SRS-2 (> 59 T). In the AS group, social symptom severity ranged from mild to severe based on the SRS-2T-scores (7.1% mild (60–65 T), 35.7% moderate (66–75 T), and 57.1% severe (≥ 76 T)). As expected, the AS group had a lower average non-verbal IQ than the NT group, including 3 autistic children who completed the WNV but obtained a non-verbal IQ < 70.

**Fig. 2:**
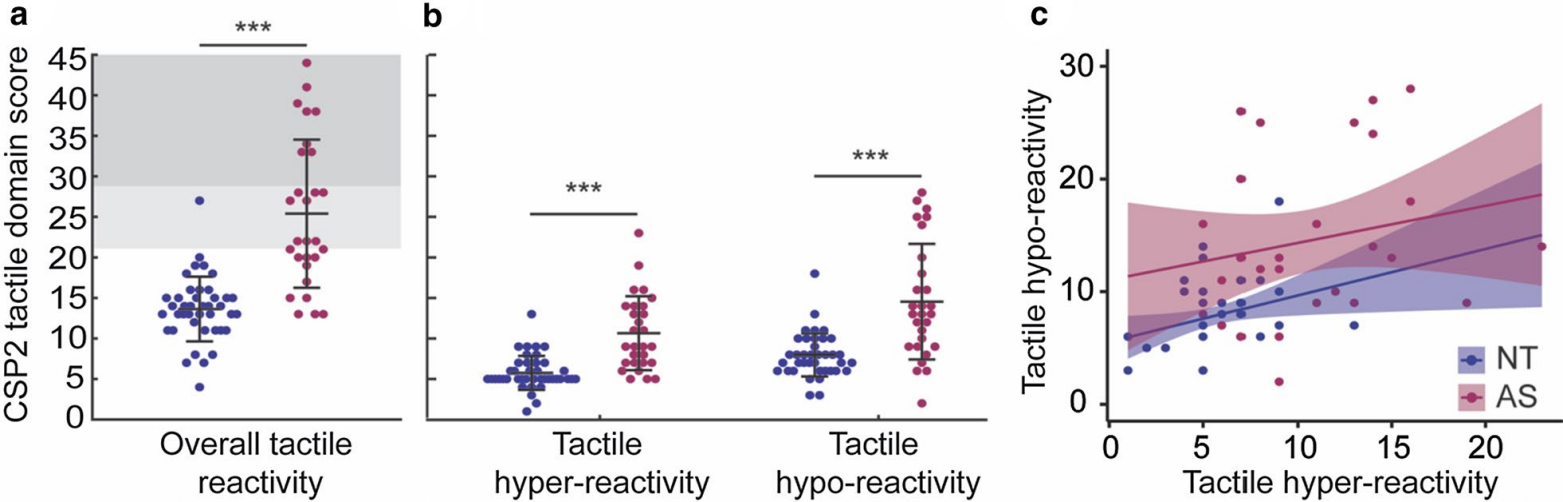
Tactile reactivity in NT and autistic children. **a** Scores on the CSP-2 tactile domain are shown for NT (blue) and autistic (wine red) children, showing greater overall tactile reactivity in autism. Light grey shading indicates ‘probable sensory differences’ and dark grey shading ‘definite sensory differences’. **b** Tactile hyper- and hypo-reactivity are shown for each group, with each of these behavioral profiles being the sum of different CSP-2 quadrants within the tactile domain. **c** Association between tactile hyper- and hypo-reactivity shown for each group (NT: *r* = 0.34, *p* = 0.028; AS: *r* = 0.21, *p* = 0.281). Dots represent individual participants (**a**, **b**, **c**) and black bars represent mean ± SD across participants (**a**, **b**). Shading indicates the 95% confidence interval on the partial correlations. Statistical group difference: ***p < 0.001

In addition, children on the autism spectrum showed significantly more parent-reported tactile reactivity (e.g., higher scores on the CSP-2 tactile domain) than the NT comparison group, as well as both greater hyper- and hypo-reactivity (Table 1, Fig. 2a, b). According to the formal cut-off for the CSP-2 tactile domain score (> 21), 43% of the autistic children fell within the normal range, 18% had ‘probable sensory differences’ (> 1 & < 2 SD, light grey area in Fig. 2a) and 39% had ‘definite differences’ (> 2 SD, dark grey area in Fig. 2a). In comparison, only 9% of NT children had ‘probable sensory differences’, while the rest fell within the normal range. Across groups, tactile hyper- and hypo-reactivity were highly correlated [*r* = 0.48, *p* < 0.001], 95% CI [0.30 0.70]]. Within groups, these two behavioral profiles were significantly associated for the NT [*r* = 0.34, *p* = 0.028], 95% CI [0.02 0.63]], but not the AS group [*r* = 0.21, *p* = 0.281], 95% CI [− 0.11 0.56]] (Fig. 2c).

### Somatosensory potentials and adaptation

**Fig. 3:**
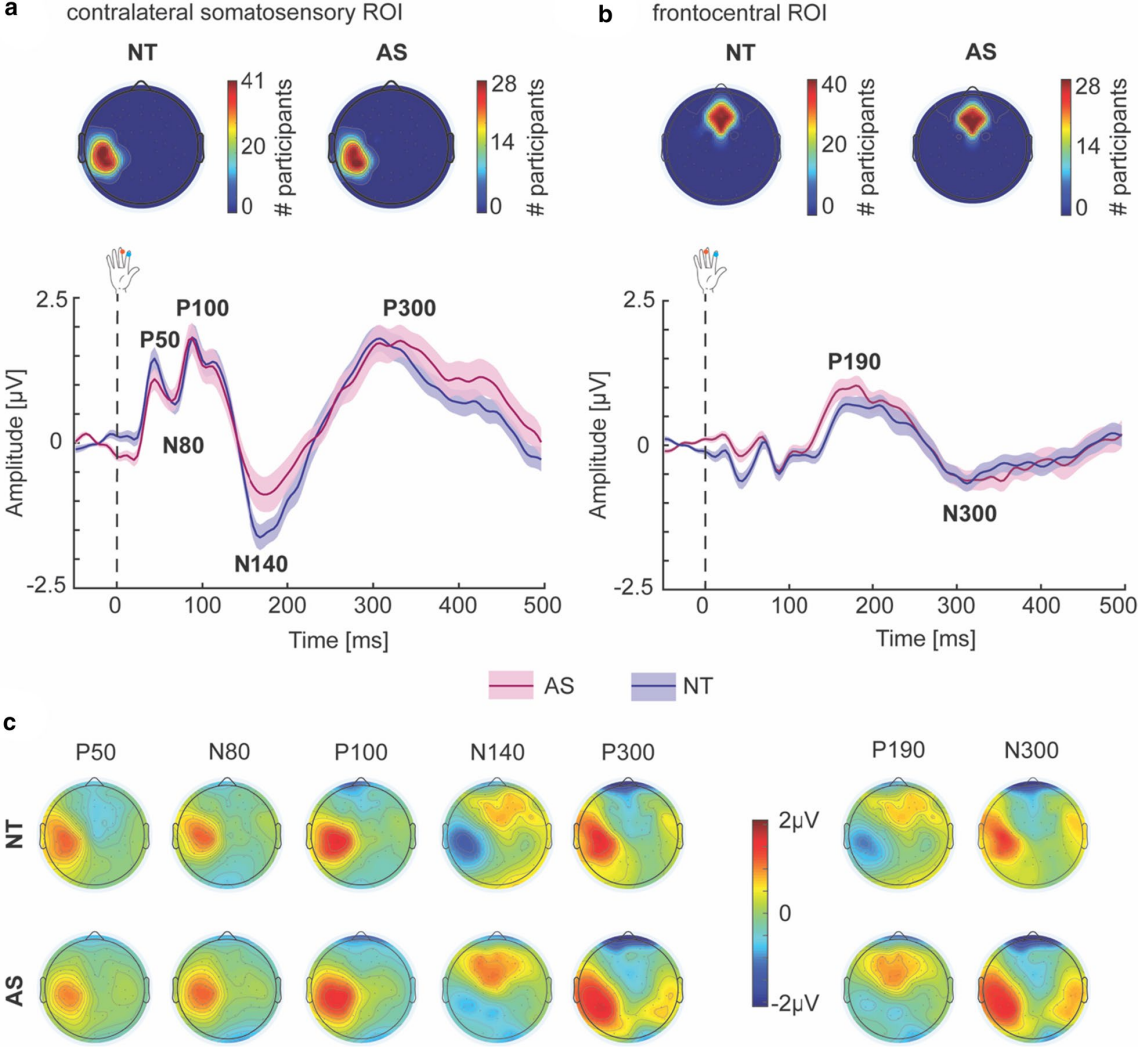
SEPs to passive tactile stimulation. **a, b** Individual ROIs over contralateral somatosensory cortex and frontocentral region were selected for each participant (top). The topographical plots show the overlap of selected electrodes for each group, with the color bar representing the number of participants for which that electrode (area) was selected. Grand-averaged SEP traces in response to passive tactile stimulation from ROIs over the contralateral somatosensory cortex (**a**, bottom) and frontal cortex (**B,** bottom) for the NT (blue) and AS (wine red) groups for the 1050 ms ISI. Major SEP responses are clearly distinguishable: P50 (30–55 ms), N80 (55–80 ms), P100 (80–125 ms), N140 (150 –210 ms), P300 (270–350 ms) over somatosensory cortex as well as P190 (150–240 ms) and N300 (280–400 ms) over bilateral frontal cortex. Dashed line at time 0 ms indicates time of fingertip stimulation. **c**, Topographical plots representing neural activity averaged over the respective time windows for each SEP response show a predominantly contralateral somatosensory area activation, with frontocentral areas being activated during later processing stages. Shaded area (**a**, **b**) indicates between-participant SEM

Grand-averaged SEP traces in response to passive tactile stimulation to the right fingers are shown in Fig. 3a (bottom) for the NT and AS groups, using the 1050 ms ISI. The early and mid-latency (P50, N80, P100) and later SEP responses (N140, P300) from contralateral somatosensory cortex are identifiable, with inverted polarity across the frontocentral scalp region (Fig. 3b, bottom). Over the bilateral frontal cortex, two later responses (P190, N300) are identifiable, reflecting the propagation of tactile information from posterior to anterior. The gross morphology and time course of the SEPs are highly similar between AS and NT groups. Individual ROIs were selected for each participant, with Fig. 3a, b (top panel) showing the overlap of selected electrodes for each group.

#### Early and mid-latency SEPs

The first SEP response peaked ~ 45 ms after stimulation over the contralateral somatosensory cortex, followed by a negative response at ~ 70 ms and a major positive peak at ~ 90 ms (Fig. 3a, c). Analysis of peak latency of these early SEP responses did not reveal a significant difference between AS and NT groups (F-statistics and *p* values of all ANCOVAs are summarized in Table 2). Similarly, analysis of mean amplitude revealed no group differences for the P50, N80 and P100 components over the contralateral somatosensory cortex.

**Table 2:** ANCOVA results for differences in SEP responses between NT and AS groups

	Peak latency	Mean amplitude
*Contralateral somatosensory ROI*		
P50	*F*_(1,65)_ = 0.06, *p* = 0.801, *η*^2^ = 0.001 [0 0.06]	*F*_(1,65)_ = 1.73, *p* = 0.193, *η*^2^ = 0.026 [0 0.14]
N80	*F*_(1,65)_ = 0.39, *p* = 0.533, *η*^2^ = 0.006 [0 0.09]	*F*_(1,65)_ = 0.10, *p* = 0.753, *η*^2^ = 0.002 [0 0.07]
P100	*F*_(1,65)_ = 1.60, *p* = 0.210, *η*^2^ = 0.024 [0 0.13]	*F*_(1,65)_ = 0.00, *p* = 0.994, *η*^2^ = 0.000 [0 0]
N140	***F***_**(1,64)**_** = 7.70, p = 0.007, η**^**2**^** = 0.107 [0.01 0.26]**	*F*_(1,64)_ = 5.32, *p* = 0.024*, *η*^2^ = 0.077 [0.01 0.22]
P300	*F*_(1,65)_ = 0.41, *p* = 0.525, *η*^2^ = 0.006 [0 0.09]	*F*_(1,65)_ = 0.02, *p* = 0.892, *η*^2^ = 0.000 [0 0.04]
*Frontocentral ROI*		
P190	*F*_(1,65)_ = 0.02, *p* = 0.771, *η*^2^ = 0.001 [0 0.04]	*F*_(1,65)_ = 0.54, *p* = 0.465, *η*^2^ = 0.008 [0 0.10]
N300	*F*_(1,65)_ = 0.01, *p* = 0.953, *η*^2^ = 0.001 [0 0.03]	*F*_(1,65)_ = 0.01, *p* = 0.924, *η*^2^ = 0.001 [0 0.03]

ANCOVA results controlling for age and sex. Significant effects that survived multiple comparison correction (using FDR) at *p*_corr_ < 0.05 are indicated in bold while effects at *p* < 0.05 uncorrected that did not survive multiple comparison correction are indicated by *. Effect sizes (*η*^2^ ranging between 0 and 1) and their 95% confidence intervals (CI in square brackets) are given

#### Later SEPs

For later SEP responses from contralateral somatosensory cortex, a negative response at ~ 175 ms was followed by a broad positivity ~ 300 ms post-stimulation (Fig. 3a, c). Interestingly, the N140 latency statistically differed between groups (Table 2, Additional file 1: Fig. S1) and this group difference survived multiple comparison correction (*p*_corr_ = 0.035). Specifically, young children on the autism spectrum showed a shorter N140 peak latency (172.81 ± 19.5 ms) relative to the NT group (182.65 ± 21.2 ms) and including IQ as a covariate did not change this result [*F*_(1,63)_ = 5.81, *p* = 0.019, *η*^2^ = 0.084, 95% CI of effect size [0.01 0.23]]. Similarly, a significant group difference was identified for the N140 response amplitude, with the AS group exhibiting a smaller (i.e., less negative) N140 amplitude (− 0.62 ± 1.47 μV) compared to the NT group (− 1.23 ± 1.30 μV); however, this result did not survive multiple comparison correction (*p*_corr_ = 0.120). No significant differences in peak latency and mean amplitude between AS and NT groups were found for the somatosensory P300.

In addition, two later SEP responses were identified over the bilateral frontal cortex, including a positive peak at ~ 190 ms, followed by a broad negative response at ~ 310 ms (Fig. 3b, c). Again, no significant differences in peak latency and mean amplitude between AS and NT groups were found for these frontal responses (Table 2).

#### Adaptation effect

**Fig. 4:**
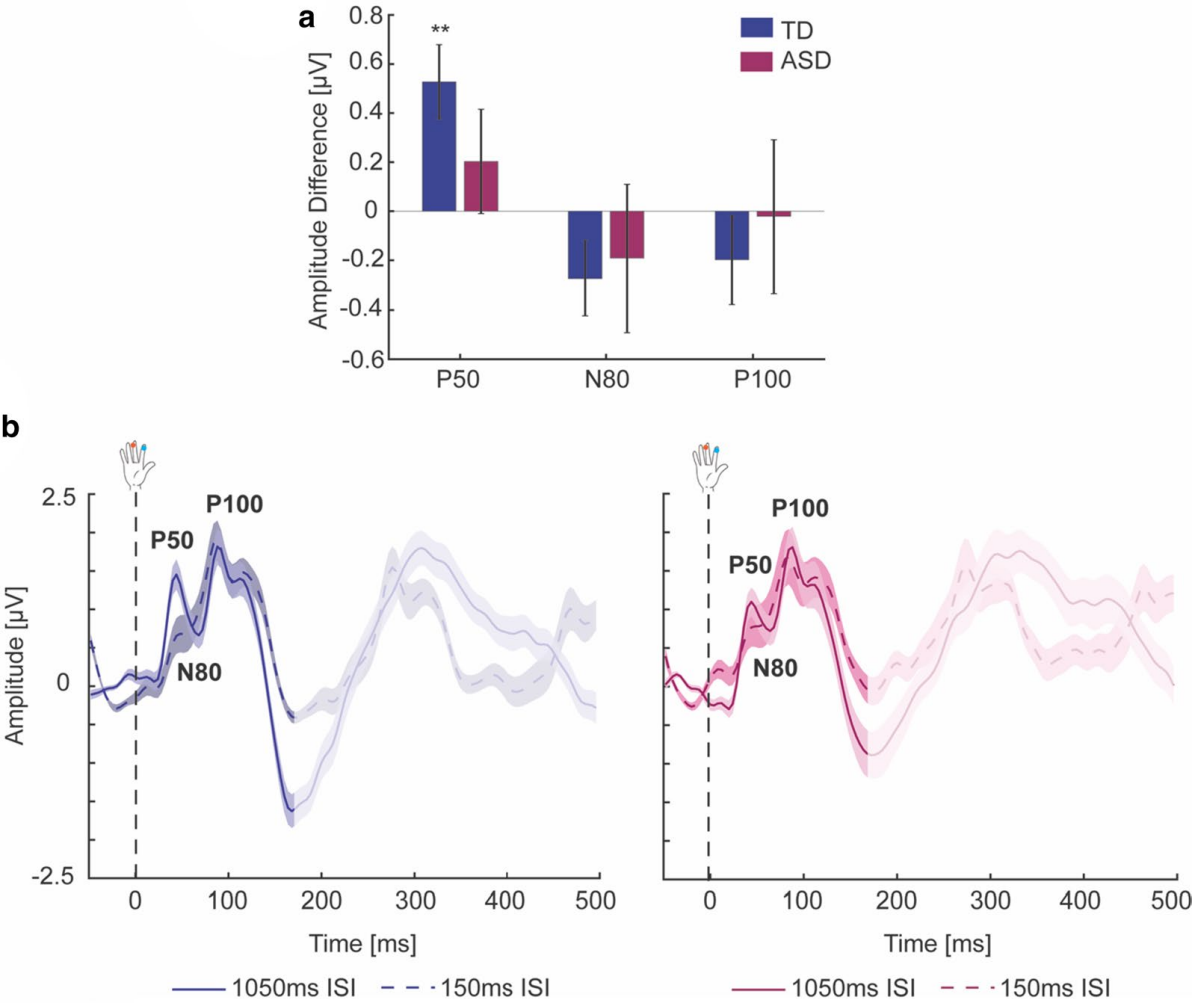
Somatosensory adaptation to repeated tactile stimulation. **a** The mean amplitude difference between long and short ISI is shown for the early and mid-latency SEP responses over the contralateral somatosensory cortex for the NT (blue) and AS (wine red) groups. Note that positive values represent greater adaptation. **b** Grand-averaged SEP traces in response to passive tactile stimulation from ROIs over the contralateral somatosensory cortex for the NT (blue) and AS (wine red) groups are shown for the long ISI (1050 ms, solid line) and the short ISI (150 ms, dashed line). The adaptation of early and mid-latency SEP responses (P50, N80, P100) can be seen as the difference between long and short ISI (see A). The time window of later SEP responses overlapped with the SEP to the subsequent stimulus in the short ISI condition and thus did not allow for an assessment of adaptation of these later responses (greyed-out portion of SEP traces). Error bars (**a**) and shaded area (**b**) represent between-participant SEM. Statistical difference from zero: **p < 0.01

Next, we compared the amount of adaptation early and mid-latency SEP responses exhibited with repetitive stimulation between AS and NT groups. A significant effect of ISI on the amplitude of the P50 was observed over the contralateral somatosensory cortex, with the short ISI leading to a significant reduction in mean amplitude across groups (F-statistics and *p* values of all ANCOVAs are summarized in Table 3, Fig. 4); however, this effect was at trend level significance after multiple comparison correction (*p*_corr_ = 0.083). There was no effect of group or interaction between these two factors, indicating that the amount of adaptation of the P50 response was statistically comparable between AS and NT groups; however, a significant reduction in mean amplitude (adaptation) was only observed in the NT group [*t*_(40)_ = 3.50, *p* = 0.002] and not in the AS group [*t*_(27)_ = 0.96, *p* = 0.352]. For the N80, a trend towards a significant amplitude difference between the short and long ISI was revealed, which did not survive multiple comparison correction, and no effect of group or interaction was present. There were no main effects (ISI, group) or interactions for the P100.

**Table 3:** ANCOVA results for differences in adaptation effect between NT and AS groups

	Group	ISI	Interaction
*Contralateral somatosensory ROI*			
P50	*F*_(1,65)_ = 0.30, *p* = 0.583, *η*^2^ = 0.005 [0 0.08]	*F*_(1,65)_ = 4.49, *p* = 0.038*, *η*^2^ = 0.065 [0 0.20]	*F*_(1,65)_ = 1.61, *p* = 0.209, *η*^2^ = 0.024 [0 0.14]
N80	*F*_(1,65)_ = 0.01, *p* = 0.921, *η*^2^ = 0.000 [0 0.03]	*F*_(1,65)_ = 3.83, *p* = 0.055*, *η*^2^ = 0.056 [0 0.19]	*F*_(1,65)_ = 0.12, *p* = 0.731, *η*^2^ = 0.002 [0 0.07]
P100	*F*_(1,65)_ = 0.13, *p* = 0.723, *η*^2^ = 0.002 [0 0.07]	*F*_(1,65)_ = 1.32, *p* = 0.255, *η*^2^ = 0.020 [0 0.13]	*F*_(1,65)_ = 0.34, *p* = 0.565, *η*^2^ = 0.005 [0 0.09]

ANCOVA results controlling for age and sex. Effects at *p* < 0.05 uncorrected that did not survive multiple comparison correction (using FDR) are indicated by *. Effect sizes (*η*^2^ ranging between 0 and 1) and their 95% confidence intervals (CI in square brackets) are given

### Associations between somatosensory potentials and tactile reactivity

**Fig. 5:**
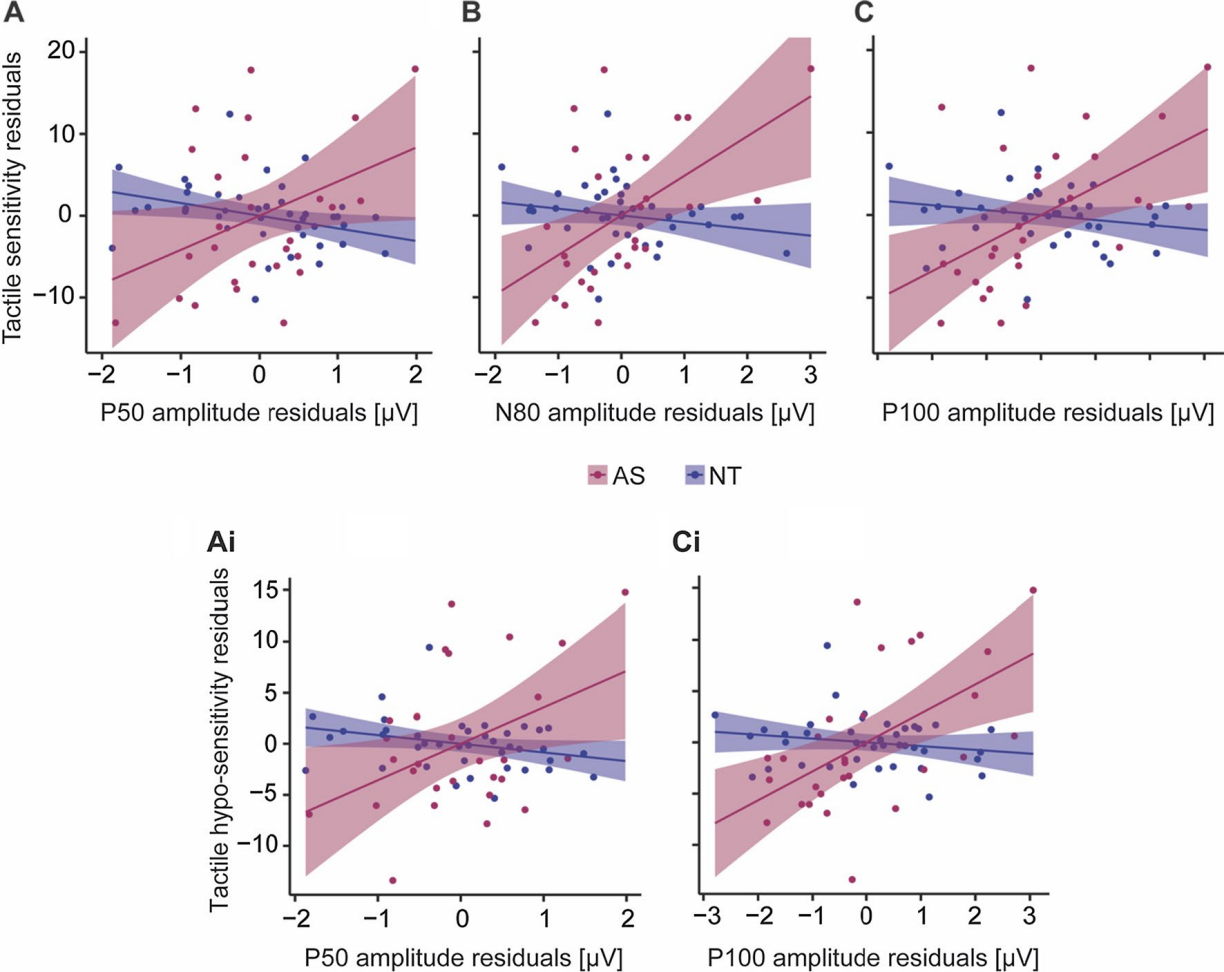
Associations between tactile reactivity measures and SEP responses. Partial correlations are shown for significant group by tactile reactivity interactions found in the general linear models. Positive partial correlations were observed between overall tactile reactivity and P50 (**a**), N80 (**b**), and P100 (**C**) amplitude for the AS group [P50: *r* = 0.38, *p* = 0.057; N80: *r* = 0.53, *p* = 0.005; P100: *r* = 0.52, *p* = 0.007], but negative (mostly non-significant) correlations for the NT group [P50: *r* = − 0.35, *p* = 0.029; N80: *r* = − 0.21, *p* = 0.208; P100: *r* = − 0.17, *p* = 0.297]. Similarly**,** positive partial correlations were observed between tactile hypo-reactivity specifically and P50 (**ai**) and P100 (**ci**) amplitude for the AS group [P50: *r* = 0.42, *p* = 0.032; P100: *r* = 0.60, *p* = 0.002], who showed the opposite pattern to the NT group [P50: *r* = − 0.32, *p* = 0.046; P100: *r* = − 0.21, *p* = 0.209]. All associations are controlled for age and sex. Shading indicates the 95% confidence interval on the partial correlations

Next, we investigated the relation of our neural measures (properties of SEPs and their adaptation) with parent-reported tactile reactivity. Across the whole sample, regressing overall tactile reactivity and its interaction with group for each SEP response revealed significant group differences in the associations between tactile reactivity and the amplitude of early and mid-latency SEP responses from contralateral somatosensory cortex [P50: *F*_(1,63)_ = 7.85, *p* = 0.007, effect size *η*^2^ = 0.111, 95% CI of effect size [0.01 0.26]; N80: *F*_(1,63)_ = 5.54, *p* = 0.022, effect size *η*^2^ = 0.081, 95% CI of effect size [0.01 0.23]; P100: *F*_(1,63)_ = 4.54, *p* = 0.037, effect size *η*^2^ = 0.067, 95% CI of effect size [0 0.21]]. Including IQ as covariate did not change these results [P50: *F*_(1,62)_ = 8.86, *p* = 0.004, effect size *η*^2^ = 0.125, 95% CI of effect size [0.01 0.28]; N80: *F*_(1,62)_ = 5.88, *p* = 0.018, effect size *η*^2^ = 0.087, 95% CI of effect size [0.01 0.23]; P100: *F*_(1,62)_ = 4.77, *p* = 0.033, effect size *η*^2^ = 0.071, 95% CI of effect size [0 0.21]]; however, only the group difference in the association between tactile reactivity and the P50 response survived multiple comparison correction (*p*_corr_ = 0.035), while the other effects were trending (both *p*_corr_ = 0.06). Specifically, NT children with a greater P50 response amplitude showed less tactile reactivity [partial correlation: *r* = − 0.35, *p* = 0.029, 95% CI [− 0.61 − 0.12]], whereas autistic children showed a trend towards the opposite pattern, with greater P50 response amplitude related to more tactile reactivity [partial correlation: *r* = 0.38, *p* = 0.057, 95% CI [− 0.19 0.75]] (Fig. 5a). For the N80 and P100 components, a smaller (i.e., less negative) and greater response amplitude respectively, was related to more tactile reactivity in autistic children [N80 partial correlation: *r* = 0.53, *p* = 0.005, 95% CI [0.11 0.83]; P100 partial correlation: *r* = 0.52, *p* = 0.007, 95% CI [0.11 0.82]], but not NT children [N80 partial correlation: *r* = − 0.21, *p* = 0.208, 95% CI [− 0.46 0.13]; P100 partial correlation: *r* = − 0.17, *p* = 0.297, 95% CI [− 0.47 0.13]] (Fig. 5b, c). There were no group differences in associations between tactile reactivity and adaptation of SEP responses.

To gain further insight into the relation between tactile reactivity and neural responses to touch, we next separately regressed tactile hyper-reactivity and hypo-reactivity and their interaction with group for each SEP response. There were significant group differences in the associations between tactile hypo-reactivity and the P50 [*F*_(1,63)_ = 5.75, *p* = 0.019, effect size *η*^2^ = 0.084, 95% CI of effect size [0.01 0.23]] and P100 [*F*_(1,63)_ = 4.56, *p* = 0.037, effect size *η*^2^ = 0.067, 95% CI of effect size [0 0.21]] amplitude from contralateral somatosensory cortex, which were at trend level significance after multiple comparison correction (both *p*_corr_ = 0.09). Again, NT children with a greater P50 response amplitude showed less tactile hypo-reactivity [partial correlation: *r* = − 0.32, *p* = 0.046, 95% CI [− 0.57, − 0.06]], whereas autistic children showed the opposite pattern, with greater P50 response amplitude relating to more tactile hypo-reactivity [partial correlation: *r* = 0.42, *p* = 0.032, 95% CI [0.06, 0.69]] (Fig. 5ai). Similarly, a greater P100 response amplitude was related with more tactile hypo-reactivity in autistic [partial correlation: *r* = 0.60, *p* = 0.002, 95% CI [0.08, 0.79]], but not NT children [partial correlation: *r* = − 0.21, *p* = 0.209, 95% CI [− 0.47, 0.76]] (Fig. 5ci). There were no group differences in associations between tactile hyper-reactivity and neural responses to touch.

### Exploratory analysis based on tactile reactivity

**Fig. 6:**
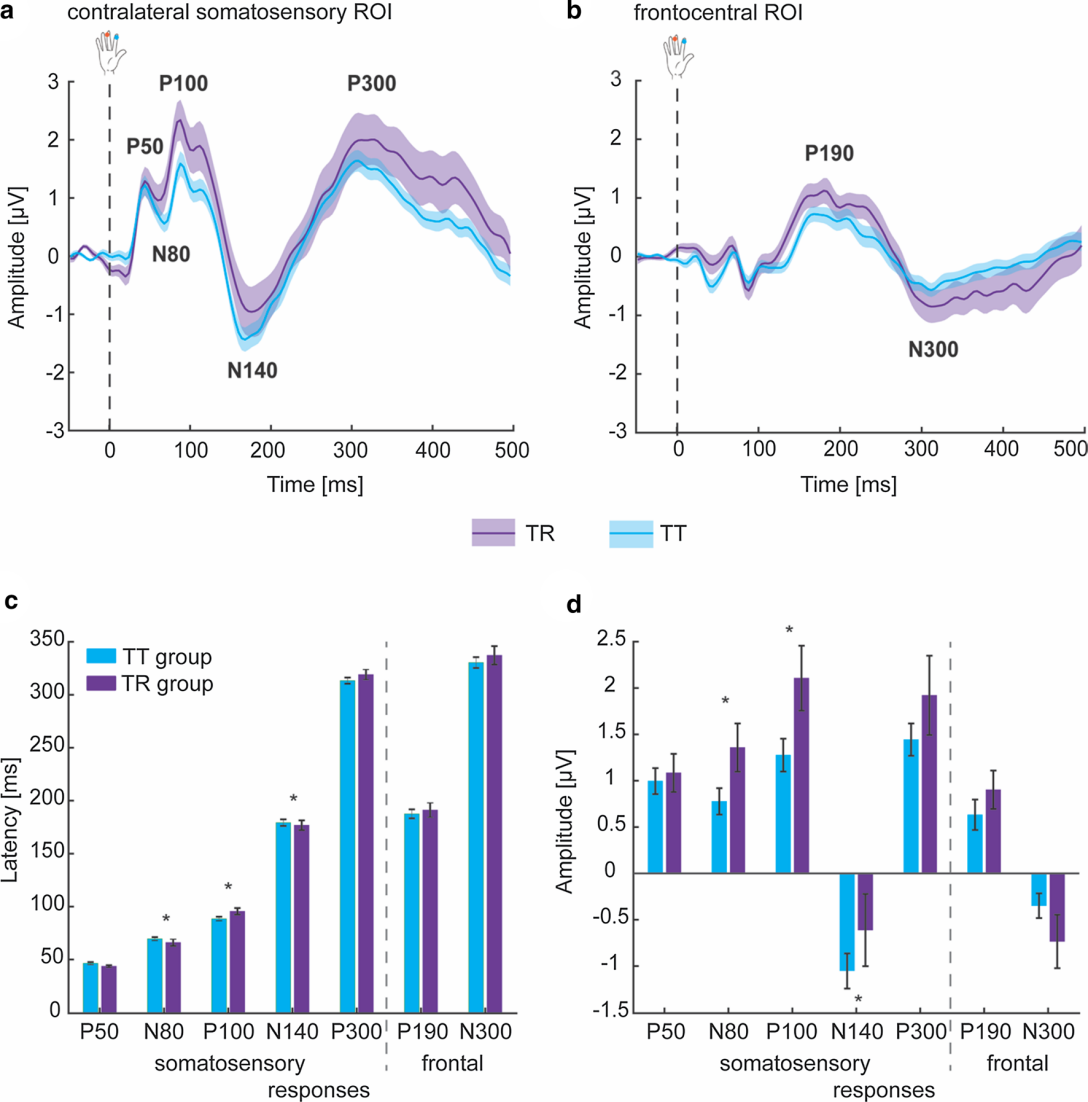
SEPs for children with and without tactile reactivity, independent of diagnosis. Grand-averaged SEP traces in response to passive tactile stimulation from ROIs over the contralateral somatosensory cortex (**a**) and frontal cortex (**b**) for the tactile typical (TT, light blue) and tactile reactive (TR, purple) groups, independent of diagnosis, for the 1050 ms ISI. Major SEP responses are clearly distinguishable: P50 (30–55 ms), N80 (55–80 ms), P100 (80–125 ms), N140 (150 –210 ms), P300 (270–350 ms) over somatosensory cortex as well as P190 (150–240 ms) and N300 (280–400 ms) over bilateral frontal cortex. Dashed line at time 0 ms indicates time of fingertip stimulation. **c**, Average latency and amplitude of SEP responses for children with and without tactile reactivity. Error bars indicate between-participant SEM. * differences between tactile typical (TT) and tactile reactive (TR) groups at *p* < 0.05 uncorrected that do not survive multiple comparison correction

Lastly, we explored whether grouping individuals based on tactile reactivity rather than clinical autism diagnosis would be informative in elucidating neurophysiological differences. To this end, all participants were regrouped based on parent-reported tactile reactivity into tactile typical (TT) and tactile reactive (TR) groups, according to the formal cut-off for the CSP-2 tactile domain (score: 21) (Fig. 6a, b). One NT child was thus moved to the tactile reactive group (*N* = 17), and twelve autistic children were moved to the tactile typical group (*n* = 49). With this sensory-based regrouping, we again observed differences in the N140 response (Fig. 6c, Table 4) such that the tactile reactive group exhibited an earlier and smaller (i.e., less negative) N140 response (peak latency: 176.84 ± 18.76 ms; mean amplitude: − 0.60 ± 1.60 μV) compared to the tactile typical group (peak latency: 179.23 ± 21.60 ms; mean amplitude: − 1.05 ± 1.24 μV). Including IQ (TR group: 89.4 ± 29.8; TT group: 100.9 ± 27.5; *t*_(64)_ = 1.47, *p* = 0.147) as a covariate did not change these results [peak latency: *F*_(1,60)_ = 4.09, *p* = 0.048, *η*^2^ = 0.064, 95% CI of effect size [0 0.21]; mean amplitude: *F*_(1,60)_ = 3.98, *p* = 0.050, *η*^2^ = 0.062, 95% CI of effect size [0 0.20]]. However, these results did not survive multiple comparison correction (peak latency: *p*_corr_ = 0.087, mean amplitude: *p*_corr_ = 0.088). In addition, we found differences in the earlier N80 and P100 responses. Specifically, we observed an earlier and higher (i.e., less negative) N80 response in the tactile reactive group (peak latency: 66.05 ± 13.03 ms; mean amplitude: 1.36 ± 1.07 μV) compared to the tactile typical group (peak latency: 69.53 ± 11.60 ms; mean amplitude: 0.77 ± 0.99 μV). For the P100, the tactile reactive grouped showed a delayed and higher response (peak latency: 95.54 ± 12.87 ms; mean amplitude: 2.11 ± 1.44 μV) compared to the tactile typical group (peak latency: 88.52 ± 13.21 ms; mean amplitude: 1.28 ± 1.24 μV). However, none of these results survived multiple comparison correction (N80 and P100 peak latency: *p*_corr_ = 0.087, N80 and P100 mean amplitude: *p*_corr_ = 0.088).

**Table 4:** ANCOVA results for differences in SEP responses between tactile reactive and tactile typical groups

	Peak latency	Mean amplitude
*Contralateral somatosensory ROI*		
P50	*F*_(1,62)_ = 1.15, *p* = 0.289, *η*^2^ = 0.018 [0 0.13]	*F*_(1,62)_ = 0.04, *p* = 0.837, *η*^2^ = 0.001 [0 0.06]
N80	*F*_(1,61)_ = 4.42, *p* = 0.040*, *η*^2^ = 0.068 [0 0.21]	*F*_(1,62)_ = 3.88, *p* = 0.053*, *η*^2^ = 0.059 [0 0.20]
P100	*F*_(1,62)_ = 3.93, *p* = 0.052*, *η*^2^ = 0.060 [0 0.20]	*F*_(1,62)_ = 5.04, *p* = 0.028*, *η*^2^ = 0.075 [0 0.22]
N140	*F*_(1,61)_ = 4.52, *p* = 0.038*, *η*^2^ = 0.069 [0 0.21]	*F*_(1,61)_ = 4.18, *p* = 0.045*, *η*^2^ = 0.064 [0 0.20]
P300	*F*_(1,61)_ = 0.98, *p* = 0.327, *η*^2^ = 0.016 [0 0.12]	*F*_(1,62)_ = 1.42, *p* = 0.238, *η*^2^ = 0.022 [0 0.13]
*Frontocentral ROI*		
P190	*F*_(1,62)_ = 0.20, *p* = 0.657, *η*^2^ = 0.003 [0 0.08]	*F*_(1,62)_ = 0.95, *p* = 0.334, *η*^2^ = 0.015 [0 0.12]
N300	*F*_(1,62)_ = 2.91, *p* = 0.093, *η*^2^ = 0.045 [0 0.17]	*F*_(1,62)_ = 1.56, *p* = 0.217, *η*^2^ = 0.024 [0 0.40]

ANCOVA results controlling for age and sex. Effects at *p* < 0.05 uncorrected that did not survive multiple comparison correction (using FDR) are indicated by *. Effect sizes (*η*^2^ ranging between 0 and 1) and their 95% confidence interval (CI in square brackets) are given

There were no significant differences in peak latency and mean amplitude between the tactile reactive and tactile typical groups for any other somatosensory responses nor adaptation effects (all *p* > 0.2). While these exploratory analyses did not survive correction for multiple comparisons, they suggest that there is utility in investigating where individuals fall on a symptom continuum that extends into both autistic and neurotypical populations rather than just diagnosis. Our results suggest that the symptom-level, distinct from diagnosis, may be an important predictor of electrophysiological responses that future studies with greater power could attempt to unravel.

## Discussion

The present study investigated tactile cortical responses in young children aged 3–6 years on the autism spectrum compared to NT children, and explored whether these neural responses relate to parent-reported tactile reactivity. Despite elevated tactile reactivity in autism, including greater hyper- and hypo-reactivity, our findings indicated no differences in the early and mid-latency stages of tactile cortical processing nor adaptation between autistic and NT children. However, later processing of tactile stimulation differed between young children on the autism spectrum and NT children. We also found that somatosensory responses during the early stages of tactile processing were associated with parent-reported tactile reactivity in autism, suggesting that greater tactile reactivity may result from enhanced neural responsiveness to touch in the early cortical processing stages.

Previous research investigating neural responses to tactile stimulation in children with autism older than 6 years has yielded mixed results, with some finding reduced [40, 60] or normal evoked responses [18, 30, 42, 51], while others observed enhanced responses [47, 50, 64]. Here, we studied early childhood (3–6 years) and found no differences in the early stages of tactile cortical processing between young children with and without autism. Similarly, we did not find a significant difference in adaptation between young autistic children and NT children, although it should be noted that a significant amount of adaptation was only observed in the neurotypical children. As no other studies have investigated tactile processing in autism at such a young age, one potential explanation for these results may be that alterations in tactile cortical processing emerge only later in development [40, 50, 60]. While this may seem counterintuitive given the early emergence of tactile difficulties in autism [53, 61], including our autistic sample, it is in line with research on early brain development in autism suggesting that young children on the autism spectrum interact differently with their environment, which may affect their brain development [26, 27]. Specifically, tactile difficulties in autism may lead to reduced engagement with touch information throughout childhood and may contribute to disruption in development of tactile processing over time. This inference is also in line with studies suggesting that differences between autistic and NT individuals increase with age [41, 58]. Alternatively, given findings of altered adaptation in infants at elevated likelihood of autism [67], the general absence of autism-related differences in early and mid-latency SEP responses and their adaptation may be attributed to heterogeneity in the phenotypic presentation of autism [59]. Even though our sample of young children had a relatively narrow chronological age range (3–6 years), there were considerable variations in autism tactile symptomatology, which possibly obscured meaningful information.

However, we did find significant differences in later neural responses between autistic and NT children. Somewhat unexpectedly, children with autism exhibited a shorter peak latency of the N140 response (~ 10 ms) in somatosensory cortex, with the mechanism underlying this faster processing speed being unclear. At a physiological level, the shortened latency in autistic relative to NT children might indicate changes in the underlying neural circuits generating this response in the secondary somatosensory cortex [2, 73] and/or a shifted excitation-inhibition balance towards a local circuit hyper-excitability [77]. Alternatively (or concomitantly), the N140 has been associated with cognitive functions, such as attention and conscious stimulus perception [10, 33, 37, 80] and hence, one might argue that the latency abnormality could be an electrophysiological correlate of “overfocused” attention, possibly due to hyper-arousal [55, 59]. However, we note that the latency difference held even after controlling for general cognitive ability, suggesting that this finding cannot solely be explained by differences in cognitive function between populations.

Interestingly, when regrouping individuals based on tactile reactivity, we continue to find latency and amplitude differences for the N140 response (e.g., shorter and less negative in the tactile reactive group), but also earlier responses between the tactile reactive and the tactile typical group. Although these exploratory analyses using tactile reactivity as a grouping variable did not survive correction for multiple comparisons, they lend support to the idea of considering where individuals fall on the spectrum of a behavioral trait, rather than a specific diagnosis, to identify unique neurophysiological differences [63].

A small number of studies have reported links between somatosensory responses and tactile features in children and adolescents with autism [18, 60]. We also found associations between our neural measures and parent-reported “real-world” reactions to touch in early childhood, with autistic and NT children showing opposite patterns. In autistic children, an enhanced early neural response (specifically the P50) related to more tactile reactivity, while it was associated with less tactile reactivity in NT children. The association in the NT group should be interpreted cautiously, however, since tactile reactivity was infrequent (e.g., less variance) in the NT group (9%) relative to the AS (57%) group. Since tactile reactivity has a significant impact on the quality of life of children with autism and their families, a better understanding of the neurophysiological mechanisms underlying this autism feature is crucial. The observed brain-behavior association in the AS group suggests that low-level somatosensory cortex processing measures may be early markers and, in the clinical context, may offer targets for therapeutic interventions. However, the specificity of this neurophysiological observation to autism is unclear. While tactile reactivity is frequent in autism, it is also relatively common in other disorders with altered sensory function, such as ADHD [12, 71] and Tourette’s syndrome [25, 70]. Hence, further work is needed to examine whether the association between early neural responses to touch and tactile reactivity is autism-specific or generalizes across disorders with altered tactile processing.

Of note, enhanced neural responsiveness to touch seemed to be related to hypo-reactivity, rather than hyper-reactivity in autism. Individuals exhibiting hyper-reactivity show exaggerated or avoidant reactions to sensory stimuli (e.g., distress to grooming activities or wearing clothes), while hypo-reactivity is characterised by an absence of or diminished/delayed reaction to sensory stimuli (e.g., unaware or slow to respond to touch or pain) [11]. Although the association between neural responsiveness and hypo-reactivity fell short of corrected significance, it initially appears paradoxical. However, it might not be surprising given that electrophysiological and parent-report measures of sensory differences likely measure complementary but not necessarily related phenomena [81, 92]. This has important implications for therapies as both the neural and behavioral targets should be considered when addressing sensory difficulties in autism. This finding also lends some support to the idea that the opposing behavioral profile of hyper- and hypo-reactivity do not share a common underlying neural mechanism [18]. In our study, these behavioral profiles were highly correlated across the entire sample, but not within the AS group, who show clinical levels of these symptoms, further supporting the divergent mechanism assertion.

In general, there is a paucity of neuroimaging studies on individuals with autism with intellectual and verbal disabilities, due to methodological challenges stemming from participants’ difficulties tolerating research protocols [46, 82]. In this study, we thus purposefully employed a passive tactile EEG task that did not require semantic and pragmatic comprehension nor an overt response to the tactile stimulation. Further, we individually tailored the testing environment and employed behavioral strategies to increase compliance. As a result, we were able to collect EEG data from > 80% of children with autism (> 95% of NT children), including those with low cognitive and/or verbal abilities (non-verbal IQ range of 49–144). Although, this approach increases sample heterogeneity, it is in line with recent commentaries calling for the inclusion of historically understudied populations within autism [46], and offers a pathway towards more generalizable conclusions about autism.

## Limitations

Several limitations of the study have to be recognized. The current study predominantly focused on the somatosensory cortex, due to its crucial role in tactile information processing [28]. However, other systems, including attentional and emotional responses to tactile input warrant further exploration (e.g., using multimodal approaches) as it is likely that multiple related processes lead to differences in tactile function in autism. One must also consider that brief tactile stimuli to the glabrous skin provide only discriminative touch information, but not affective and rewarding properties of touch. C tactile (CT) afferents, which innervate hairy skin, are thought to be related to affective responses to tactile stimuli and pain [62, 66] and might be involved in activating brain regions implicated in autism [47, 89]. It will therefore be of crucial importance for future studies to utilise both types of touch to provide a comprehensive understanding of tactile processing in individuals on the autism spectrum. Lastly, we acknowledge that our sample size was somewhat small and may have limited the power to detect subtle group differences and associations. However, imaging data collection in young children under the age of 6 years and, in particular with neurodevelopmental disorders, remains a formidable challenge, and thus, this study serves as an important step for advancing our knowledge about the neural mechanisms underlying tactile reactivity in early childhood autism.

## Conclusions

These findings offer insight into the neurophysiological mechanisms underlying tactile reactivity in early childhood autism. Findings suggest that some neural responses during tactile processing are altered in young children with autism and that tactile phenotype may associate with neurophysiological differences. Further, the association between early neural responses and tactile reactivity suggests that accessible measurements of tactile cortical processing may be indices of tactile reactivity in young children on the autism spectrum, which may have clinical implications. Future studies in children with autism should investigate the developmental trajectory of tactile processing taking into account tactile features frequently displayed by these children.

## Supplementary Information

**Additional file 1:** **Figure S1:** Latency and amplitude measures of the N140 response. **Table S1:** ANCOVA results for differences in SEP responses between NT and AS groups, controlling for age, sex and trial retention rate. **Table S2:** ANCOVA results for differences in adaptation effect between NT and AS groups. **Table S3:** ANCOVA results for differences in SEP responses between NT and AS groups with a 100ms baseline and controlling for age and sex.

## Data Availability

The datasets generated and analysed during the current study are available from the corresponding author (S.E.) upon reasonable request.

## Abbreviations

ADHD: Attention-Deficit/Hyperactivity Disorder
ADOS: Autism Diagnostic Observation Schedule
AS: Autism Spectrum
CI: Confidence Interval
CSP-2: Child Sensory Profile 2
EEG: Electroencephalography
FDR: False Discovery Rate
GDT: Global Developmental Delay
HICCUP: Healthy Infants and Children Clinical Research Program
IC: Independent Component
ICA: Independent Component Analysis
ISI: Inter-Stimulus Interval
NT: NeuroTypical
ROI: Region Of Interest
SRS-2: Social Responsiveness Scale, Second Edition
SEP: Somatosensory-Evoked Potential
TR: Tactile Reactive
TT: Tactile Typical
WNV: Wechsler Non-Verbal Scale of Ability

## Footnotes

1 Note on terminology: To acknowledge different language preferences [49, 75], the authors have chosen to use both person-first language (e.g., ‘individuals/children with autism) and identity-first language (e.g., ‘autistic children’) interchangeably to describe people on the autism spectrum.
