# Supplementary material for: Tactile cortical responses and association with tactile reactivity in young children on the autism spectrum
Source: Mol Autism. 2021 Apr 1;12:26. doi: 10.1186/s13229-021-00435-9 (PMC8017878; doi:10.1186/s13229-021-00435-9)
Supplement: Supplementary file 1 — Additional file 1: Figure S1: Latency and amplitude measures of the N140 response. Table S1: ANCOVA results for differences in SEP responses between NT and AS groups, controlling for age, sex and trial retention rate. Table S2: ANCOVA results for differences in adaptation effect between NT and AS groups. Table S3: ANCOVA results for differences in SEP responses between NT and AS groups with a 100ms baseline and controlling for age and sex. [file 13229_2021_435_MOESM1_ESM.docx]

**Additional file**


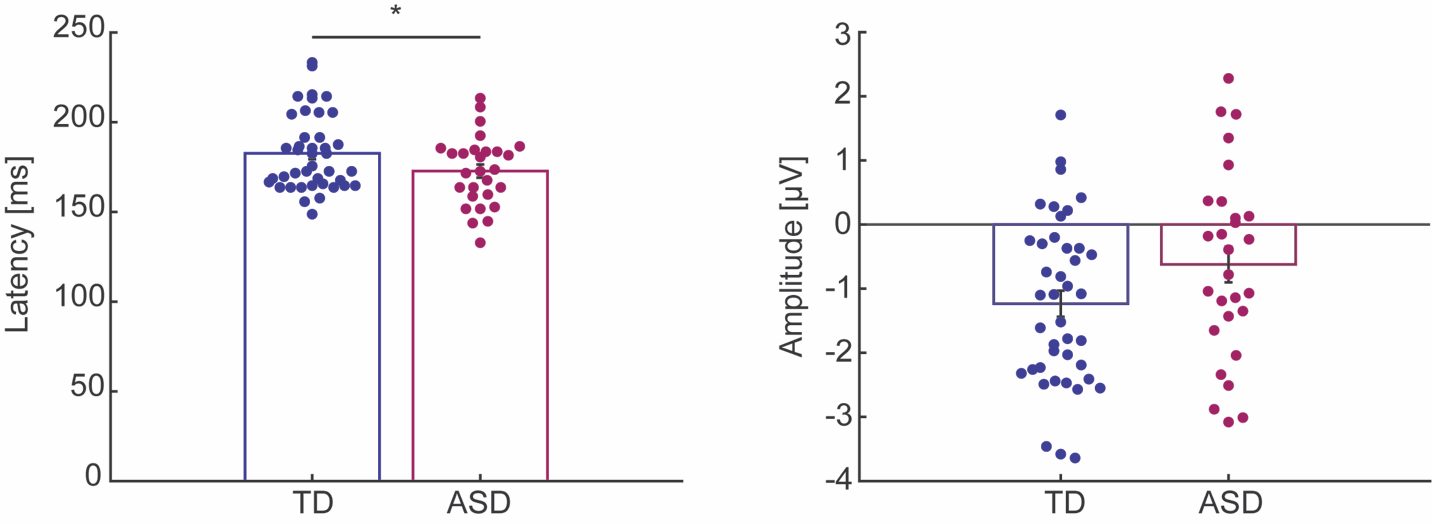


**Figure S1: Latency and amplitude measures of the N140 response.** Grand-averaged latency (left panel) and amplitude (right panel) measures for the NT (blue) and AS (wine red) groups are shown. Dots represent individual participants and black bars represent mean ±SD across participants. Significant group differences that survive multiple comparison correction at p_corr_<0.05 are indicated by *.

**Table S1: ANCOVA results for differences in SEP responses between NT and AS groups, controlling for age, sex and trial retention rate.**

|  | **Peak Latency** | **Mean Amplitude** |
| --- | --- | --- |
| **Contralateral somatosensory ROI** | |  |
| P50 | F_(1,64)_=0.04, *p*=0.849, η^2^=0.001 [0 0.03] | F_(1,64)_=0.64, *p*=0.426, η^2^=0.010 [0 0.10] |
| N80 | F_(1,64)_=0.63, *p*=0.432, η^2^=0.010 [0 0.10] | F_(1,64)_=0.94, *p*=0.337, η^2^=0.014 [0 0.12] |
| P100 | F_(1,64)_=2.29, *p*=0.100, η^2^=0.035 [0 0.16] | F_(1,64)_=0.60, *p*=0.440, η^2^=0.009 [0 0.10] |
| N140 | F_(1,63)_=5.89, *p*=0.018*, η^2^=0.086 [0.01 0.23] | F_(1,63)_=5.49, *p*=0.022*, η^2^=0.080 [0.01 0.22] |
| P300 | F_(1,64)_=0.11, *p*=0.739, η^2^=0.002 [0 0.07] | F_(1,64)_=0.77, *p*=0.385, η^2^=0.012 [0 0.11] |
| **Frontocentral ROI** | |  |
| P190 | F_(1,64)_=0.02, *p*=0.884, η^2^=0.000 [0 0.01] | F_(1,64)_=0.48, *p*=0.493, η^2^=0.007 [0 0.09] |
| N300 | F_(1,64)_=0.11, *p*=0.746, η^2^=0.002 [0 0.07] | F_(1,64)_=0.20, *p*=0.658, η^2^=0.003 [0 0.08] |
| *ANCOVA results controlling for age, sex and trial retention rate. Effects at p<0.05 uncorrected that did not survive multiple comparison correction are indicated by *. Effect sizes (*η^2^ *ranging between 0 and 1) and their 95% confidence intervals (CI in square brackets) are given.* | | |

**Table S2: ANCOVA results for differences in adaptation effect between NT and AS groups.**

|  | **Group** | **ISI** | **Interaction** |
| --- | --- | --- | --- |
| **Contralateral somatosensory ROI** | |  |  |
| P50 | F_(1,64)_=0.38, *p*=0.542, η^2^=0.006 [0 0.09] | F_(1,64)_=3.35, *p*=0.050*, η^2^=0.048 [0 0.18] | F_(1,64)_=0.074, *p*=0.787, η^2^=0.001 [0 0.05] |
| N80 | F_(1,64)_=0.51, *p*=0.476, η^2^=0.008 [0 0.09] | **F_(1,64)_=17.73, *p*<0.001, η^2^=0.217 [0.06 0.37]** | F_(1,64)_=0.74, *p*=0.394, η^2^=0.011 [0 0.11] |
| P100 | F_(1,64)_=0.17, *p*=0.684, η^2^=0.003 [0 0.08] | F_(1,64)_=0.01, *p*=0.931, η^2^=0.000 [0 0.01] | F_(1,64)_=0.61, *p*=0.439, η^2^=0.009 [0 0.10] |
| *ANCOVA results controlling for age, sex and trial retention rate. Significant effects that survived multiple comparison correction (using FDR) at p_corr_<0.05 are indicated in bold while effects at p<0.05 uncorrected that did not survive multiple comparison correction are indicated by *. Effect sizes (*η^2^ *ranging between 0 and 1) and their 95% confidence intervals (CI in square brackets) are given.* | | | |

**Table S3:** **ANCOVA results for differences in SEP responses between NT and AS groups with a 100ms baseline and controlling for age and sex.**

|  | **Peak Latency** | **Mean Amplitude** |
| --- | --- | --- |
| **Contralateral somatosensory ROI** | |  |
| P50 | F_(1,65)_=0.06, *p*=0.801, η^2^=0.001 | F_(1,65)_=0.83, *p*=0.113, η^2^=0.038 |
| N80 | F_(1,65)_=0.39, *p*=0.533, η^2^=0.006 | F_(1,65)_=0.43, *p*=0.517, η^2^=0.007 |
| P100 | F_(1,65)_=1.60, *p*=0.210, η^2^=0.024 | F_(1,65)_=0.02, *p*=0.900, η^2^=0.000 |
| N140 | **F_(1,64)_=7.70, *p*=0.007, η^2^=0.107** | F_(1,64)_=5.04, *p*=0.028*, η^2^=0.073 |
| P300 | F_(1,65)_=0.41, *p*=0.525, η^2^=0.006 | F_(1,65)_=0.14, *p*=0.707, η^2^=0.002 |
| *ANCOVA results controlling for age and sex. Significant effects that survived multiple comparison correction (using FDR) at p_corr_<0.05 are indicated in bold while effects at p<0.05 uncorrected that did not survive multiple comparison correction are indicated by *. Effect sizes (*η^2^ *ranging between 0 and 1).* | | |
